# Mutational signatures in colon cancer

**DOI:** 10.1186/s13104-019-4820-0

**Published:** 2019-12-03

**Authors:** Priyatama Pandey, Zhi Yang, Darryl Shibata, Paul Marjoram, Kimberly D. Siegmund

**Affiliations:** 1 Department of Preventive Medicine, Keck School of Medicine of the University of Southern California, 2001 N. Soto Street, Los Angeles, CA 90032 USA; 2 Department of Pathology, Keck School of Medicine of the University of Southern California, 2011 Zonal Ave, Los Angeles, CA 90033 USA

**Keywords:** Somatic mutations, Mutational process, Topic model, Latent Dirichlet allocation model

## Abstract

**Objective:**

Recently, many tumor sequencing studies have inferred and reported on mutational signatures, short nucleotide patterns at which particular somatic base substitutions appear more often. A number of signatures reflect biological processes in the patient and factors associated with cancer risk. Our goal is to infer mutational signatures appearing in colon cancer, a cancer for which environmental risk factors vary by cancer subtype, and compare the signatures to those in adult stem cells from normal colon. We also compare the mutational signatures to others in the literature.

**Results:**

We apply a probabilistic mutation signature model to somatic mutations previously reported for six adult normal colon stem cells and 431 colon adenocarcinomas. We infer six mutational signatures in colon cancer, four being specific to tumors with hypermutation. Just two signatures explained the majority of mutations in the small number of normal aging colon samples. All six signatures are independently identified in a series of 295 Chinese colorectal cancers.

## Introduction

The first large study of somatic mutations in cancer identified 20 mutational signatures in 7042 primary tumors from 30 different classes [1]. They defined mutational signatures by patterns of three consecutive nucleotides, including one base 3$^\prime$ and one 5$^\prime$ of the nucleotide substitution, and represented by a linear combination of the 96-possible three-base patterns. The mutational signatures were annotated and published in the Catalogue of Somatic Mutations in Cancer (COSMIC) database [2]. Four signatures were identified in 557 colorectal cancers [1], three signatures with probable associations attributed to one of the mechanisms of aging, DNA mismatch repair, or Pol $\epsilon$ mutation and the fourth of unknown origin.

A simple probabilistic model for mutational signatures, proposed shortly thereafter, assumed independent contributions (i.e., multiplicative probabilities) of the neighboring bases composing the nucleotide pattern [3]. This resulted in a more parsimonious model with fewer parameters and the ability to detect longer five-base signature patterns. A reanalysis of the same colon cancer data using this new probabilistic model also reported four mutational signatures, but their make-up was different. The previous Pol $\epsilon$ signature was split into two signatures, one favoring $\rm{C}>\rm{T}$ mutations at TpCpG and the second favoring $\rm{C}>\rm{A}$ at TpTpCpT, a signature four bases in length. The remaining two signatures were attributed to aging, and unknown origin. Interestingly, the DNA mismatch repair signature was not reported.

Today, the number of single-base substitution signatures in the COSMIC database has increased to 49; seven of these signatures relate to DNA mismatch-repair (MMR) deficiency. Recent studies characterizing cancers with hypermutation [4] and cancers along the gastrointestinal tract [5, 6] reported multiple MMR signatures. A recent reanalysis of data from the Cancer Genome Atlas by Liu et al. identified six signatures in colon cancer [6], four of which are identified as occurring primarily in cancers with high mutational burden. We sought to understand the connection between these six mutational signatures and those found using the probability mutational signature model.

In addition to studying the variation in mutational signatures appearing in different subtypes of colon cancers, we investigated whether the mutational signatures differed across different time periods. We classified somatic mutations by their time of occurrence, occurring in the original tumor cell (‘trunk’ mutation) or appearing *de* *novo* during tumor growth (‘branch’ mutation), and compared their signatures to those found in adult stem cells from normal colon. We exploit publicly available data from a study of adult stem cells (ASCs) in normal colon [7], the Cancer Genome Atlas (TCGA), and the International Cancer Genomics Consortium (ICGC). Our analysis identifies six mutational signatures using ASCs and TCGA colon cancers that are validated in the ICGC Chinese colorectal cancers.

## Main text

### Data

#### Human adult stem cells (ASCs) from normal colon

Whole genome sequencing of 21 samples from 6 human ASCs from normal colon was performed and published in [7]. Processed somatic mutation data were downloaded from [8].

#### TCGA colon adenocarcinoma (COAD-US)

We downloaded somatic mutation data from 435 colon adenocarcinoma from the Genomic Data Commons Data Portal [9]. The tumor characteristic microsatellite instability (high, low, stable) was downloaded as part of the clinical data. A total of 431 samples with somatic mutation data had information on microsatellite instability. We obtained the variable on Pol $\epsilon$ mutation from the supplementary data in [10]. We note that our downloading and filtering of the TCGA data resulted in notable differences from the previously analyzed data made available in [1, 3].

We classified mutations by their time of occurrence (trunk/branch) by applying the criteria of Williams et al. [11], using information on tumor purity and allele frequency. We restricted our data set to the COAD-US samples in [11] with purity $\ge$ 70% (n = 99), and classified the mutations with frequency $\ge$ 0.25 as trunk and the rest as branch. After mutation classification, six samples with fewer than 10 mutations along with their tumor-matched sample were omitted from further analysis.

#### Colorectal adenocarcinoma in China (COCA-CN)

The somatic mutation data in Chinese colorectal adenocarcinoma were downloaded from the ICGC Data Portal [12]. This data set contains 2,941,990 mutations in 295 Chinese colorectal samples.

See Additional file 1 for details on mutation filtering.

### Statistical methods

We applied the probabilistic mutation signature model [3] to infer mutation signatures and their exposure frequencies in normal colon ASCs and COAD-US tumor samples. We restricted all samples to mutations on chromosomes 1–22 and fit the model using the **pmsignature** package in R [3]. We specify the model for a five-base context and include the direction of the transcription strand (positive/negative). The four nucleotides flanking the substitution, two upstream and two downstream, are extracted from the reference genome. As the ASCs from normal colon and COAD-US samples were sequenced at different times and mapped to different reference genomes, flanking bases are extracted using the same reference to which the corresponding sample was mapped, (hg19 for ASC samples and hg38 for COAD-US). We selected the optimum number of latent mutational signatures by minimizing the Bayesian Information Criterion (BIC) and the bootstrap standard errors for the model parameters [3].

The Shiny app iMutSig [13] was used to compare our discovered signatures with the published mutational signatures from pmsignature and from the COSMIC mutational signature website [2, 3]. iMutSig uses cosine similarity to compute the similarity of any two mutational signatures. When comparing our five-base signature to the three-base signature in COSMIC, we sum the probabilities of the signature vector from the five-base model over the features unmeasured in the three-base model. Due to the independence assumption of our model, this is equivalent to a comparison using just the features shared in common by the two models.

Finally, we applied a hierarchical latent Dirichlet allocation model (HiLDA) [14] to test the equivalence of mutational signature exposures between trunk and branch mutations. We used the posterior distributions of the mean differences to test for differential exposures for any single signature (signature-level tests). The analysis was performed in R using the *HiLDA* package.

### Results

Mutational signature analysis was applied to 127,748 mutations from 431 COAD-US samples and 860 mutations from 6 normal colon ASCs. The highest numbers of somatic mutations are found in the MMR-deficient, MSI-H and Pol $\epsilon$ cancers (Additional file 1: Figure S1). We fit the probability mutation signature model for different numbers of mutational signatures (2 through 8) and using the criteria of low bootstrap error and low BIC, selected six mutational signatures as having the best fit (Additional file 1: Figure S2).

**Fig. 1:**
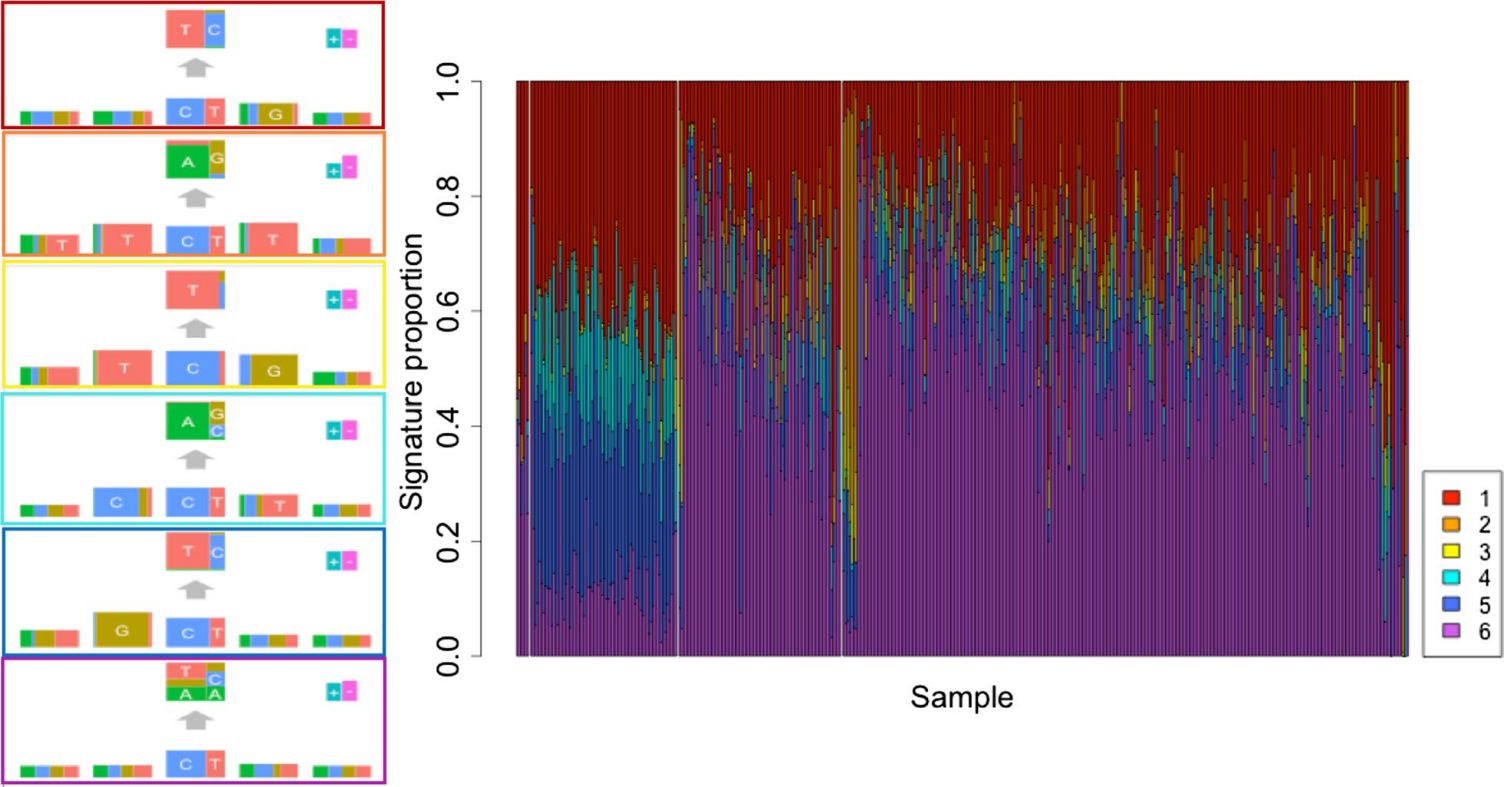
Signatures and their estimated mutational exposures for normal ASCs and COAD-US tumors. Six mutational signatures estimated from 6 Normals and 431 COAD-US tumors (72 MSI-H, 80 MSI-L, and 279 MSS). Estimated mutational signatures (left) and signature mutational exposures (right), ordered as follows: Normal, MSI-H, MSI-L and MSS. In the figures to the left, each mutation feature is represented by a rectangle with colored area proportional to the expected frequency of each nucleotide. The more unequal the 4 nucleotide frequencies, the taller the rectangle. The five columns represent positions − 2, − 1, 0, 1, 2, relative to the single-base substitution. The upper right rectangles represent the expected frequency of the two transcription strands (+/−). Each signature is named by the color of the box enclosing it. These are ordered from top to bottom: red, orange, yellow, cyan, blue, purple. To the right, each vertical bar represents a tumor, and the colors indicate the relative frequency of that mutational signature in the tumor. The order of colors from top to bottom match the order of colors on the left (red to purple)

Figure 1 shows the six inferred mutational signatures along with the estimates of signature mutational exposures. The six signatures included the four signatures previously identified by Shiraishi et al. [3] (red, orange, yellow, purple). The red signature was described as being due to aging, whereas the orange and yellow signatures were described as being due to the deregulated activity of the polymerase Pol $\epsilon$, while purple was of unknown origin. Two additional mutational signatures (cyan and blue, Fig. 1) were inferred to occur most frequently in MSI-H tumors, the blue signature also appearing in tumors with deregulated activity of the polymerase Pol $\epsilon$. Deregulated polymerase activity is defined using mutational data (see [10]). The cyan signature reported a $\mathrm {C}>\mathrm {A}$ substitution occurring with a 5$^\prime$ C; the blue signature identified $\mathrm {C}>\mathrm {T}$ and $\mathrm {T}>\mathrm {C}$ substitutions occurring with a 5$^\prime$ G (Fig. 1). Both of these signatures resemble signatures previously reported by Shiraishi et al. [3] in stomach cancer (pmsignatures 11 and 27 with cosine similarities of 0.79 and 0.88, respectively, Table 1). The six normal ASC and MMR-proficient tumor mutation catalogs were composed primarily of the red and purple signatures. For more on these samples see Additional file 1.

**Table 1:** Cosine similarities of de-novo signatures (6 signatures in Fig. 1) with the COSMIC (May 2019) single-base substitution signatures, and with the pmSignatures from Shiraishi’s paper

De-novo	COSMIC							pmSignature					
Signatures	SBS1	SBS6	SBS10a	SBS10b	SBS15	SBS20	SBS40	1	7	8	11	15	27
Red	0.830	0.778	0.020	0.238	0.530	0.214	0.281	0.002	0.863	0.215	0.317	0.305	0.034
Orange	0.002	0.014	0.943	0.260	0.050	0.087	0.353	0.991	0.013	0.015	0.002	0.139	0.152
Yellow	0.261	0.207	0.006	0.914	0.102	0.025	0.069	0.001	0.289	0.971	0.052	0.084	0.001
Cyan	0.004	0.042	0.108	0.041	0.116	0.884	0.279	0.175	0.024	0.002	0.005	0.173	0.876
Blue	0.267	0.737	0.036	0.108	0.844	0.250	0.204	0.005	0.462	0.139	0.791	0.485	0.028
Purple	0.157	0.354	0.270	0.263	0.322	0.415	0.911	0.225	0.379	0.153	0.159	0.815	0.292

We compared our new signatures to those found in the COSMIC v89 May 2019 database (Mutational Signatures v3) (Table 1). Our blue signature resembles COSMIC signature SBS15, associated with defective DNA mismatch repair (cosine similarity 0.844). The new cyan signature resembles SBS20, reported to be associated with combined deficiencies in DNA mismatch repair and *POLD*1 proofreading (cosine similarity 0.884).

**Fig. 2:**
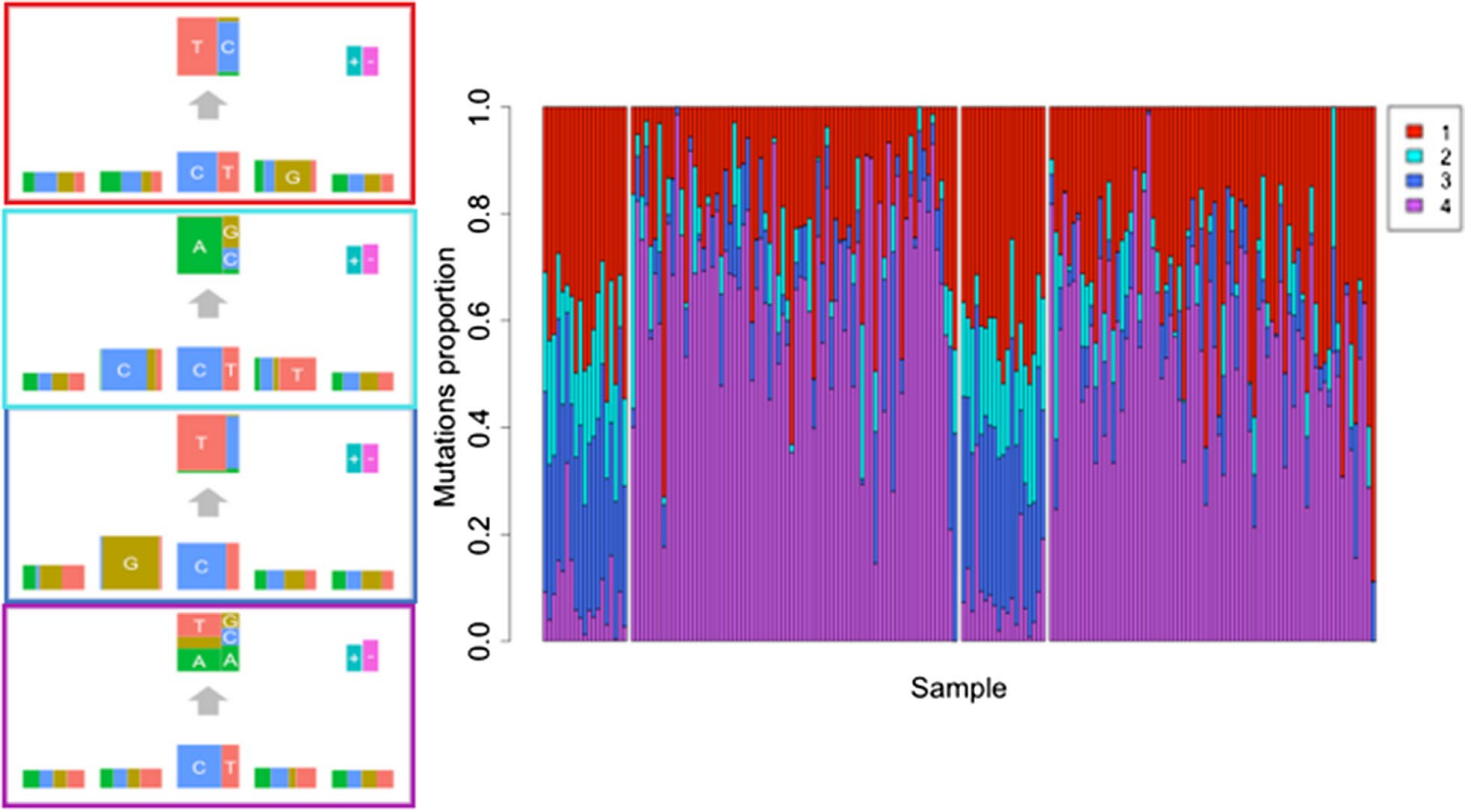
Branch–Trunk Signatures and their mutational exposures in COAD-US tumors. Four mutational signatures estimated from 186 samples of branch and trunk mutations from 93 COAD-US tumors. Estimated mutational signatures (left) and signature mutational exposures (right), ordered as follows: MSI-H branch, nonMSI-H branch, MSI-H trunk, nonMSI-H trunk. For more details see legend to Fig. 1

To investigate whether the signatures we detected in the tumors varied by the time of occurrence, we refitted the mutational signature model to the subset of 93 tumors with mutations grouped separately as trunk or branch. We specified and estimated four signatures only, as none of the 93 tumors carried the Pol $\epsilon$ signatures. The results in Fig. 2 show little discernible difference in mutational signature burden between trunk and branch mutations. Indeed, the MSI tumors show no evidence of differential trunk/branch mutational burden (all signature-specific 95% credible intervals include zero) (Additional file 1: Table S2). Interestingly, the MSS tumors show a 9.6% higher mutational exposure of the red signature ($\mathrm {C}>\mathrm {T}$ at CpG) in trunk compared to branch mutations (95% credible interval: 0.047–0.114).

Finally, we sought to replicate our mutational signatures in an independent set of cancers from China. We apply the same probabilistic mutation signature model to the Chinese COCA-CN data set and identify the same six mutational signatures (Additional file 1: Figures S3, S4), replicating those extracted from the COAD-US data set. Although we lack information on tumor subtype, when ordering the tumors by the total number of mutations, a correlate for the MSI-H subtype, the pattern of estimated burdens for each mutational signature mimics those from the analysis of COAD-US cancers (see Additional file 1: Methods for details).

### Discussion

We conducted a mutational signature analysis of colon adenocarcinomas from TCGA. We identified six mutational signatures using the probabilistic mutational signature model with five-base patterns, whereas an early publication only reported four [3]. The ASCs from normal colon and MMR-proficient tumors showed a mutational signature for aging, whereas the MMR-deficient tumors showed multiple MMR-related signatures.

A recent paper by Liu et al. also reported six signatures but allowed only three-base patterns in a more highly parameterized model [6]. The signatures from the two approaches were slightly different. Our model pooled substitutions with similar neighboring bases into a single signature (e.g. GpC > GpT and GpT > GpC in Fig. 1, blue) when theirs did not. Conversely, theirs combined substitutions with different neighboring bases into a single signature (CpC > CpA and GpC > GpT in COSMIC signature SBS6) when ours did not. The signatures we found replicated in an independent set of Chinese COCA-CN samples.

After classifying our mutations into time of occurrence, trunk or branch, we found the signature for aging (red) was more frequent in trunk than branch mutations from MSS tumors but the same was not true for MSI tumors. This replicates the results from an earlier study of MSS colon cancers that also found a higher mutational exposure of the aging signature in trunk compared to branch mutations [14]. The lack of any new mutational signature in branch mutations, despite the different micro-environments of cancer from normal colon, is interesting.

## Limitations

- TCGA published high-quality mutations from their Multi-Center Mutation Calling in Multiple Cancers (MC3) project in March 2018 [15], after the data for this paper were downloaded. The MC3 project reported variants on 389 (90%) of our 431 cancers, identifying 104,557 (82%) of the mutations we used for those same tumors. They identified 240585 variants, 1.9 times the number in our study. The smaller number of mutations in our analysis likely affected the precision of our estimates, and potentially also our sensitivity to detect new signatures. This limitation could be more problematic for the analysis of trunk versus branch mutations as we are likely to be differentially missing more branch than trunk mutations.
- The somatic mutation data from the Chinese COCA-CN samples did not include variant allele frequency so we were unable to filter this data set using the same strict rules. Nevertheless, we still found evidence for the same six signatures in colon cancer, and the burdens of the new signatures in MSI-H tumors were over-represented in the tumors with high mutation burden. Therefore, despite not having information on microsatellite instability of the cancer, we can roughly infer which tumors they are based on their mutational signatures and total mutation burden. This remains to be validated.
- Our new analysis discovered a signature with a preponderance of $\mathrm {C}>\mathrm {A}$ substitutions, a common substitution for smoking, occurring at CpC sites. This signature appears in MSI-H tumors more frequently than MSS tumors. At the same time, epidemiologic research has found that a history of smoking is more frequent in patients with MSI-H compared to MSS tumors [16, 17]. Unfortunately, we do not have information on smoking history for COAD-US patients to investigate this.

## Supplementary information

**Additional file 1.** Additional figures and tables.

## Data Availability

Only publicly available data were analyzed in this paper. The final datasets and code are available from the corresponding author upon request.

## Abbreviations

AIC: Akaike Information Criterion
BIC: Bayesian Information Criterion
COAD-US: Colon Adenocarcinoma US
COSMIC: Catalogue of Somatic Mutations in Cancer
ICGC: International Cancer Genomics Consortium
MMR: mismatch repair
MSI-H: microsatellite instable high
MSI-L: microsatellite instable low
MSS: microsatellite stable
TCGA: The Cancer Genome Atlas
