# Supplementary material for: Mutational signatures in colon cancer
Source: BMC Res Notes. 2019 Dec 3;12:788. doi: 10.1186/s13104-019-4820-0 (PMC6889194; doi:10.1186/s13104-019-4820-0)
Supplement: Supplementary file 1 — Additional file 1. Additional figures and tables. [file 13104_2019_4820_MOESM1_ESM.pdf]

**Additional Files**

Additional file 1 — Additional Results

Methods

*Human Adult Stem Cells (ASCs) from Normal Colon*

In order to analyze these data jointly with the whole exome data from the cancers, we selected the subset of mutations in exonic regions using the target regions of the nextera exome kit bed file and combined the samples from the same donor to give us six ASC samples in total. The filtering of somatic mutations and pooling across samples from the same donor had negligible effect on the mutational signatures estimated from an analysis of the ASC samples.

*TCGA Colon Adenocarcinoma (COAD-US)*

Since TCGA used MuTect to identify somatic mutations, we applied the filtering criteria recommended for MuTect to reduce the number of false-positive calls (minimum read depth of 14 in the cancer and allele fraction of 0.10) [1]. We also required three or more variant alleles, an additional filter to reduce false-positives used by Williams et al. [3].

*Colorectal Adenocarcinoma in China (COCA-CN)*

For the purpose of data visualization, we predicted Pol $\epsilon$ mutation and microsatellite instability for the unannotated cancers from the somatic mutation data. Pol $\epsilon$ mutation was predicted from the estimated mutational signatures. We estimated the mutational signatures in the COCA-CN samples using the model determined to fit the Normal ASC and COAD-US data best. We classified the tumor as a Pol $\epsilon$ mutation carrier if $>$50% of the signature fractions were attributed to the two Pol $\epsilon$ signatures (Figure 1, orange and yellow signatures). The remaining tumors were classified as MSI-H or MSI-L/MSS based on the total number of somatic mutations. We created a barplot of the number of somatic mutations, ordering the tumors from largest to smallest, left to right. We then computed the difference in number of somatic mutations between adjacent samples (step height), and classified all tumors to the left of the largest step as MSI-H and all to the right as MSI-L/MSS. These inferred labels were used for plotting the data in Figure S3.

Results

Table S1 lists the average of the estimated mutational signature exposures according to sample type (normal ASC, MSI-H, Pol $\epsilon$, MSI-L, MSS). The normal ASCs and MMR proficient cancers are dominated by the red and purple signatures - the red signature representing the C$>$T substitution occurring primarily at CpG sites and the purple signature capturing the substitutions that do not favor any particular flanking bases. MSI-H tumors are dominated by four signatures (red, cyan, blue, purple), and Pol $\epsilon$ by three signatures (orange, yellow, blue) with only modest fractional contributions from the red and purple signatures. However, if we consider the numbers of mutations in the red signature, the signature likely due to aging, we find an average of 328 in MSI-H, 214 in Pol $\epsilon$, 20 in MSI-L and 19 in MSS, showing that the numbers of mutations from the red signature are higher, on average, in Pol $\epsilon$ tumors than in both the MSI-L and MSS cancers. The normal ASC samples had an average of 23 mutations attributed to the red signature, which resembles the numbers in both MSI-L and MSS cancers. Whereas the orange and yellow signatures are essentially unique to the Pol $\epsilon$ cancers, appearing hardly at all (mean percentage $<$ 1-4%) in the other subtypes, the cyan signature (CC$>$CA), is essentially unique to MSI-H tumors. The blue signature appears to be a general signature of MMR deficiency, contributing to both MSI-H and Pol $\epsilon$ mutational burdens (average percentages 29% and 17%, respectively).

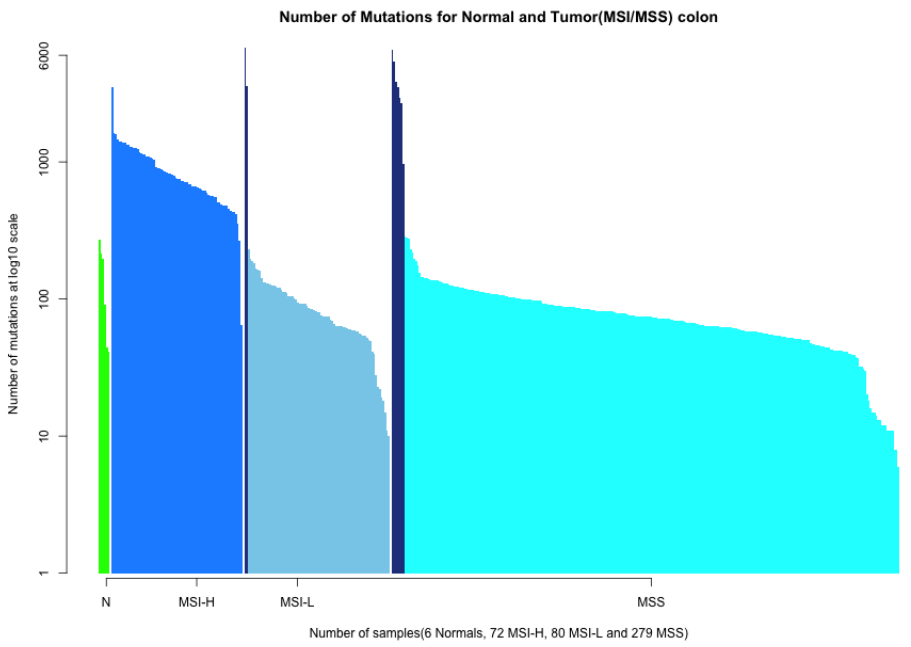


**Figure S1 Numbers of somatic mutations for normal ASCs and COAD-US tumors.** Number of single nucleotide substitutions per sample, with samples ordered by tissue type: 6 Normal adult colon stem cells (green) and 431 COAD-US tumors (72 MSI-H, blue; 80 MSI-L, sky blue; 279 MSS, cyan). Pol $\epsilon$ samples are shown in dark royal blue (2 MSI-L, 7 MSS).

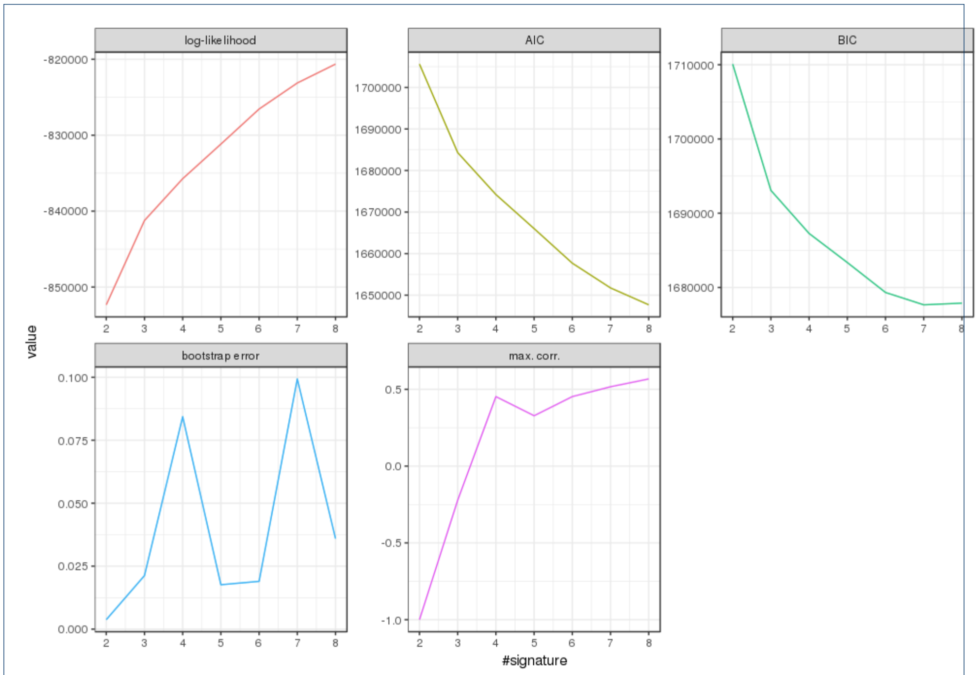


**Figure S2 Criteria for selecting the number of signatures** Log-likelihood, Akaike's information criterion (AIC), Bayesian Information criterion (BIC), bootstrap error [2], maximum correlation [2], all shown as a function of number of signatures.

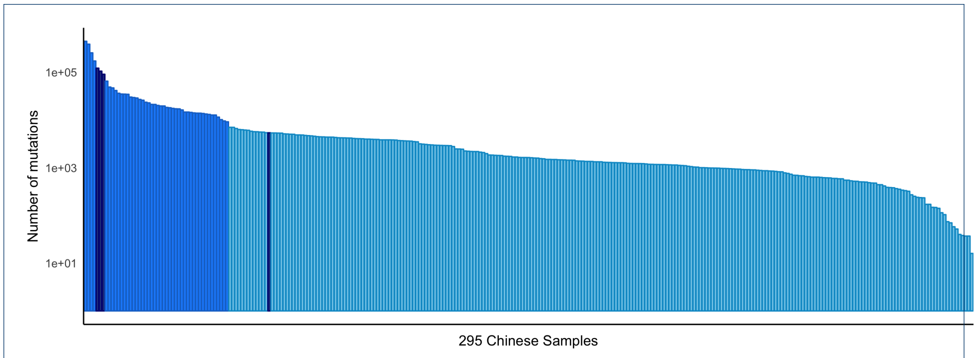


**Figure S3 Numbers of somatic mutations for ICGC COCA-CN tumors.** The numbers of somatic mutations for 295 Chinese colorectal adenocarcinomas (COCA-CN). Samples with blue are categorized as MSI-H whereas royal blue represents the Pol $\epsilon$ samples and the light blue are categorized as MSI-L/MSS.

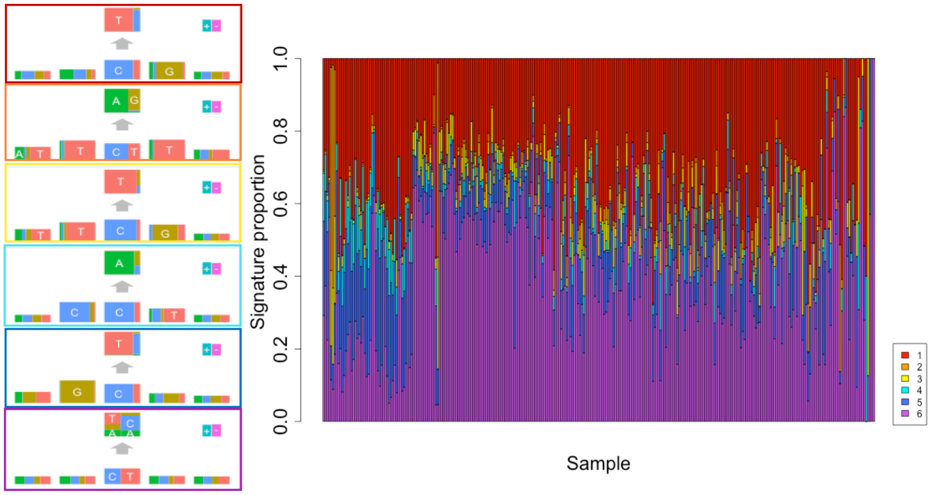


**Figure S4 Signatures and their mutational exposures for ICGC COCA-CN tumors.** Six mutational signatures estimated from 295 Chinese COCA-CN colorectal adenocarcinomas. Estimated mutational signatures (left) and signature mutational exposures for each tumor (right), with tumors ordered by the total number of somatic mutations (high to low).

**Table S1** Summary of mutational burden percentages in 6 Normal ASCs and 431 COAD-US tumors (72 MSI-H, 80 MSI-L and 279 MSS samples).

| Signature | Summary | Normal | MSI-H | Pol $\epsilon$ | MSI-L | MSS |
|---|---|---|---|---|---|---|
| Red | 1st Qu. | 51.82 | 34.37 | 4.49 | 17.16 | 18.25 |
| | Mean | 55.31 | 39.27 | 4.83 | 27.02 | 27.19 |
| | 3rd Qu. | 60.44 | 43.91 | 5.96 | 33.17 | 33.86 |
| Orange | 1st Qu. | 0.00 | 0.00 | 48.86 | 0.00 | 0.00 |
| | Mean | 0.10 | 0.48 | 54.92 | 3.31 | 3.08 |
| | 3rd Qu. | 0.00 | 0.80 | 58.66 | 5.53 | 4.77 |
| Yellow | 1st Qu. | 0.60 | 0.17 | 10.40 | 0.00 | 0.00 |
| | Mean | 2.68 | 1.07 | 15.05 | 2.52 | 2.91 |
| | 3rd Qu. | 4.61 | 1.34 | 17.72 | 3.65 | 4.46 |
| Cyan | 1st Qu. | 0.00 | 14.16 | 0.21 | 0.00 | 0.00 |
| | Mean | 0.53 | 16.63 | 1.67 | 4.35 | 3.98 |
| | 3rd Qu. | 0.29 | 19.21 | 2.44 | 6.80 | 5.68 |
| Blue | 1st Qu. | 4.74 | 26.33 | 11.58 | 4.34 | 3.01 |
| | Mean | 6.38 | 29.43 | 16.83 | 8.88 | 8.23 |
| | 3rd Qu. | 8.08 | 32.49 | 21.34 | 12.58 | 12.48 |
| Purple | 1st Qu. | 27.26 | 9.03 | 4.78 | 41.11 | 45.89 |
| | Mean | 34.99 | 13.11 | 6.71 | 53.91 | 54.61 |
| | 3rd Qu. | 39.47 | 15.20 | 6.88 | 69.76 | 64.93 |

**Table S2** The signature-specific estimates of the difference in mean mutational exposures between the trunk and branch mutations, $\Delta_{Trunk-Branch}$ in Figure 2.

| Subtype | Signature | Mean | Median | 95% Credible interval |
|---|---|---|---|---|
| MSI-H | Red | 0.019 | 0.020 | [-0.036, 0.073] |
| | Cyan | -0.005 | -0.005 | [-0.046, 0.037] |
| | Blue | -0.013 | -0.013 | [-0.063, 0.037] |
| | Purple | -0.002 | -0.002 | [-0.037, 0.033] |
| nonMSI-H | Red | 0.096 | 0.096 | [0.047, 0.144] |
| | Cyan | 0.004 | 0.003 | [-0.015, 0.025] |
| | Blue | -0.002 | -0.002 | [-0.033, 0.030] |
| | Purple | -0.098 | -0.098 | [-0.152, -0.042] |

**Author details**

**References**

1. Cibulskis K, Lawrence MS, Carter SL, Sivachenko A, Jaffe D, Sougnez C, Gabriel S, Meyerson M, Lander ES, Getz G (2013) Sensitive detection of somatic point mutations in impure and heterogeneous cancer samples. Nature biotechnology 31(3):213
2. Shiraishi Y, Tremmel G, Miyano S, Stephens M (2015) A simple model-based approach to inferring and visualizing cancer mutation signatures. PLoS genetics 11(12):e1005,657
3. Williams MJ, Werner B, Barnes CP, Graham TA, Sottoriva A (2016) Identification of neutral tumor evolution across cancer types. Nature genetics 48(3):238
